# High-Abundance Heterotrophic Bacteria Inhabit the 85° E Hydrothermal Plume of the Explosive Volcanic Zone at Gakkel Ridge, Arctic Ocean

**DOI:** 10.3390/biology14081036

**Published:** 2025-08-12

**Authors:** Juan Yu, Yejian Wang, Xiqiu Han, Hanlin Wang, Tao Zhang, Weiwei Ding, Chi Yang, Yinxia Fang, Jiabiao Li

**Affiliations:** 1 Ocean College, Zhejiang University, Zhoushan 316021, China; juanyu@zju.edu.cn; 2 State Key Laboratory of Submarine Geoscience, Second Institute of Oceanography, Ministry of Natural Resources, Hangzhou 310012, China; wanghlin@sio.org.cn (H.W.); tao_zhang@sio.org.cn (T.Z.); wwding@sio.org.cn (W.D.); aligatou@163.com (C.Y.); fangyx@sio.org.cn (Y.F.); jbli@sio.org.cn (J.L.)

**Keywords:** Gakkel ridge, hydrothermal plume, explosive volcanism, *Alcanivorax*, chemoautotrophic bacteria, carbon cycle

## Abstract

While under-ice submarine hydrothermal systems provide critical insights into extremophile adaptations, the ecological impacts of explosive volcanism on these ecosystems remain poorly constrained. We successfully detected evidence of hydrothermal activities and explosive volcanism at 85° E, the eastern volcanic zone, ultra-slow spreading Gakkel Ridge. Hydrothermal plume, surface sediments, and volcanic glass samples were systematically collected to investigate the diversity of microbial communities. Our results revealed two distinct microbial regimes in hydrothermal plume: (1) chemoautotrophic bacteria (*Sulfurimonas* and *SUP05_cluster*), prevalent in global basaltic hydrothermal systems, potentially involved in carbon fixation through the CBB and rTCA cycles and (2) *Alcanivorax* (up to 82.5%), known for degrading hydrocarbons. Sediment profiles showed a depth-dependent decline of *Alcanivorax*, tightly coupled with TOC (1.05% to 0.45%, r = 0.75, *p* < 0.05). Additionally, the *Alcanivorax* MAGs demonstrated their potential in degrading various types of organic carbon, especially in alkane degradation. Strikingly, this pattern contrasts with hydrothermal plumes from effusive volcanic zones (Aurora and Polaris regions), where *Alcanivorax* was undetectable. We speculate that the surge of *Alcanivorax* in the 85° E hydrothermal plume was associated with the violent disturbances caused by explosive volcanism. This mechanism accelerates microbial-mediated carbon turnover rates compared to a stable hydrothermal ecosystem.

## Simple Summary

Under-ice submarine volcanic-hydrothermal systems serve as a pivotal environment for investigating the limits of extremophiles and advancing our understanding of biogeochemical processes. Evidence of hydrothermal activities and explosive volcanic events was detected at 85° E on the Gakkel Ridge, Arctic Ocean. A systematic analysis of microbial diversity and metabolic potential was conducted in the hydrothermal plumes and surface sediments. The results indicated that, in addition to the prevalence of chemolithoautotrophic microbes in hydrothermal plume, a significant abundance of heterotrophic bacteria, such as *Alcanivorax*, was observed. These heterotrophic bacteria are likely involved in the decomposition of hydrocarbons and complete mineralization into CO_2_. We speculate that this phenomenon is linked to the release of sedimentary organic carbon into the water column, a consequence of the recent explosive volcanic activity.

## 1. Introduction

The Arctic Ocean, as an amplifier of global climate change, provides a distinctive and crucial setting for investigating the interactions between the lithosphere, hydrosphere, biosphere, and atmosphere [1,2]. However, the perennial ice cover of the Arctic Ocean has limited our understanding of the deep-sea mass and energy exchanges between the spheres, as well as their impacts on the ecosystem and geochemical processes. The Gakkel Ridge (approximately 1800 km long), lying beneath the Arctic Ocean, represents a critical yet understudied node in global plate tectonics, with its distinct ecological systems also remaining poorly studied [3,4,5,6]. Despite its ultra-slow spreading rate (6–13 mm/year), the Gakkel Ridge has been found to exhibit significant evidence of volcanism and hydrothermal activity during decades of exploration, showcasing unique tectono-magmatic processes [6,7,8,9,10]. In the ice-covered Arctic deep-sea environment, life forms exhibit remarkable survival strategies, thriving under extreme conditions such as frequent volcanic activity and subzero temperatures [8,11,12]. Investigating the energy acquisition and adaptive evolutionary mechanisms of microbes in this environment will contribute to understanding deep-sea biogeological puzzles, the limits of life on Earth, and will also provide potential analogs for extraterrestrial life searches, such as on Saturn’s moon Enceladus [13,14].

Deep-sea hydrothermal plumes typically maintain stable microbial networks fueled by inorganic energy sources [15,16]. Dominant groups often include Gammaproteobacteria (*SUP05*), Campylobacteria (Such as *Sulfurimonas* and *Sulfurovum*), Deltaproteobacteria (SAR324), Alphaproteobacteria (SAR11), and Thaumarchaeota (Marine Group I) [17,18,19,20,21,22]. Chemosynthetic microorganisms obtain energy by oxidizing reductive substances such as H_2_S, H_2_, CH_4_, Fe, and Mn to fix CO_2_ [16,23]. On the southern part of the Gakkel Ridge (82.5° N), the Aurora Vent Field, the only confirmed hydrothermal vent field currently (visually confirmed in 2014), exemplifies this paradigm, dominated by sulfur/hydrogen-oxidizing chemoautotrophs that drive carbon fixation [11,24]. Previous studies have investigated microbial community structures and metabolic characteristics in hydrothermal systems in relatively stable environments [25,26,27,28,29,30]. However, how local geological events, such as volcanic eruptions, influence hydrothermal microbial ecosystems remains unclear and warrants further investigation.

The 85° E segment is located in the eastern volcanic zone of the Gakkel Ridge, where explosive volcanism has been shown to occur relatively more frequently than in the western volcanic zone (where the Aurora Field was found). Edmonds et al. reported a relatively thick nonbuoyant hydrothermal plume (up to 1400 m) at the 85° E segment [8,31,32,33]. However, it remains unclear whether the structure, energy acquisition, and adaptive evolutionary mechanisms of the microbial communities at the 85° E segment are similar to those of the Aurora Field. We hypothesize that local geological environments or events, such as volcanic eruptions, may shape or disrupt microbial communities and hydrothermal ecosystems.

During the Joint Arctic Scientific Middle-Ocean Ridge Insight Expedition (JASMInE) in 2021, hydrothermal plume anomalies were detected, as well as seafloor hydrothermal microbial mats and volcanic ash, at the 85° E segment of the Gakkel Ridge. We systematically collected hydrothermal plume and surface sediments samples to analyze the microbial diversity, metabolic potential and their relationships with environmental factors, testing the hypothesis that explosive volcanism shapes distinct microbial community structures.

## 2. Materials and Methods

### 2.1. Hydrothermal Plume Detection and Near-Bottom Optical Survey

**Figure 1:**
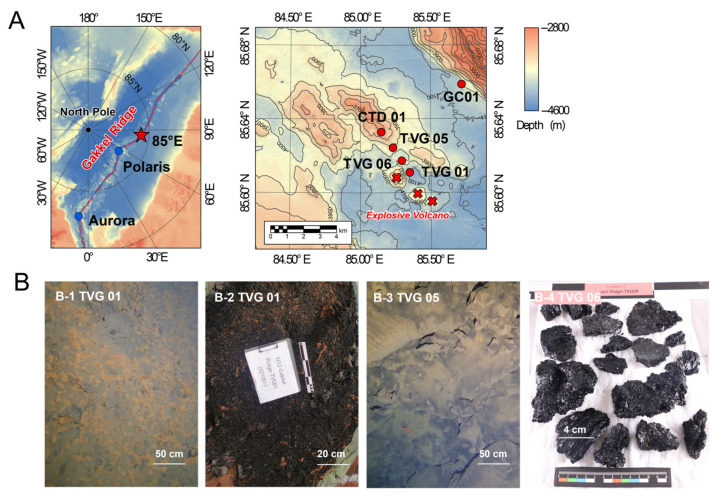
Sampling sites and seafloor observations at the 85°E segment, Gakkel Ridge. (**A**) Location of sampling stations. (**B**) Seafloor observations and samples on board, (**B**-**1**) microbial mats observed by TV-Grab at TVG 01 station; (**B**-**2**) volcanic glass chips grabbed at TVG 01 station. The thickness of volcaniclastic deposits exceeds 10 cm; (**B**-**3**) explosive volcanic ash observed at TVG 05 station; (**B**-**4**) fresh volcanic glass fragments recovered at TVG 06 station.

**Figure 2:**
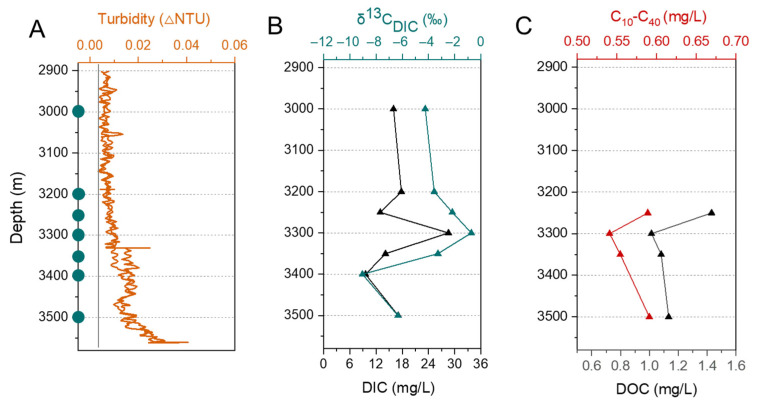
Environmental parameters in 85° E hydrothermal plume and background seawater (**A**–**C**). (**A**) Turbidity values, with blue filled circles on the Y-axis representing the depths at which samples were taken. (**B**) DIC concentrations and δ^13^C_DIC_. (**C**) DOC and C_10_–C_40_ concentrations.

Hydrothermal plume signals were detected at CTD 01 station (85.63° N, 85.14° E) on the Gakkel Ridge during JASMInE aboard the icebreaker “Xuelong 2” in August 2021 (Figure 1A). The SBE-32 carousel water sampler, integrated with conductivity-temperature-depth (CTD) profilers and turbidity sensor for detecting hydrothermal plume anomalies. The turbidity profiles revealed the presence of two anomaly layers: one at depths between 3300 and 3500 m, and another below 3500 m (not reaching the seafloor). The peak anomalies occurred at approximately 3400 m (0.02 NTU) and 3550 m (0.03 NTU) after background subtraction (Figure 2A). The near-bottom optical survey (TV-Grab) showed the presence of volcanic ash and hydrothermal microbial mats at stations TVG 05 and TVG 01, situated at approximately 1 km and 3 km away from CTD 01 station, respectively (Figure 1B).

### 2.2. Sampling and Physicochemical Analysis

**Figure 3:**
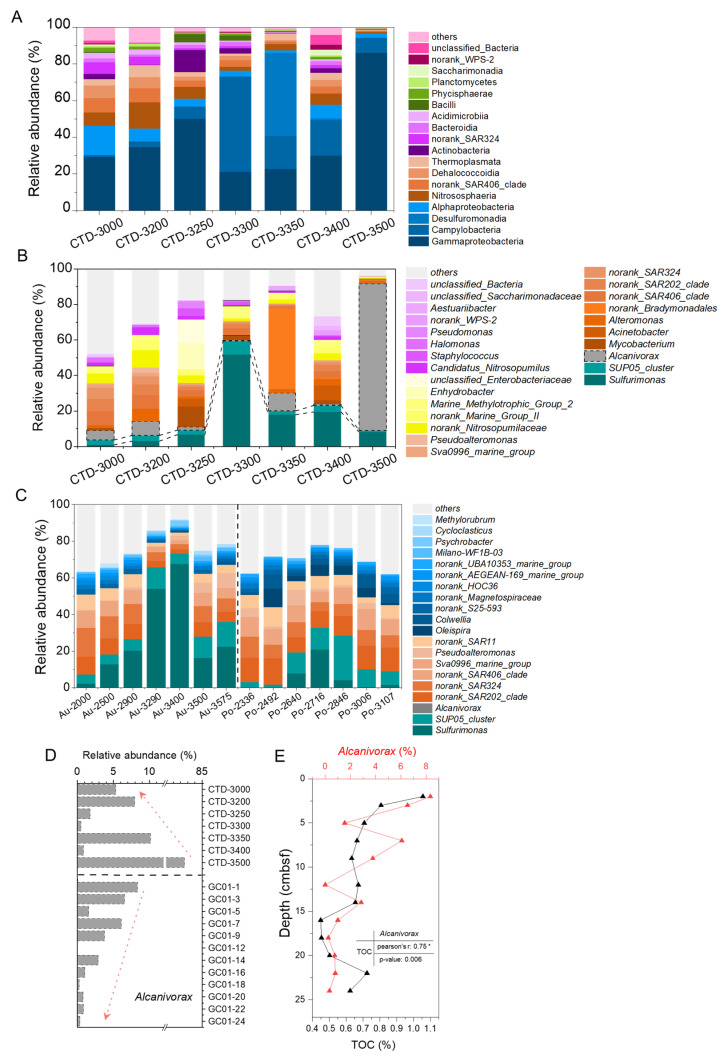
Microbial community composition at class level (**A**) and genus level (**B**–**D**). (**A**) 85° E samples, relative abundances below 1% were classified as “others”. (**B**) 85° E samples, taxa with relative abundances below 2% were classified as “others“; (**C**) Aurora and Polaris hydrothermal plume samples, taxa with relative abundances below 2% were classified as “others“; (**D**) Relative abundance of *Alcanivorax* in hydrothermal plume (CTD 01) and surface sediments (GC 01) at 85° E area. (**E**) The abundance of *Alcanivorax* and the content of TOC in sediments, and their correlation, * is the Pearson correlation coefficient was statistically significant at the 0.05 level.

**Figure 4:**
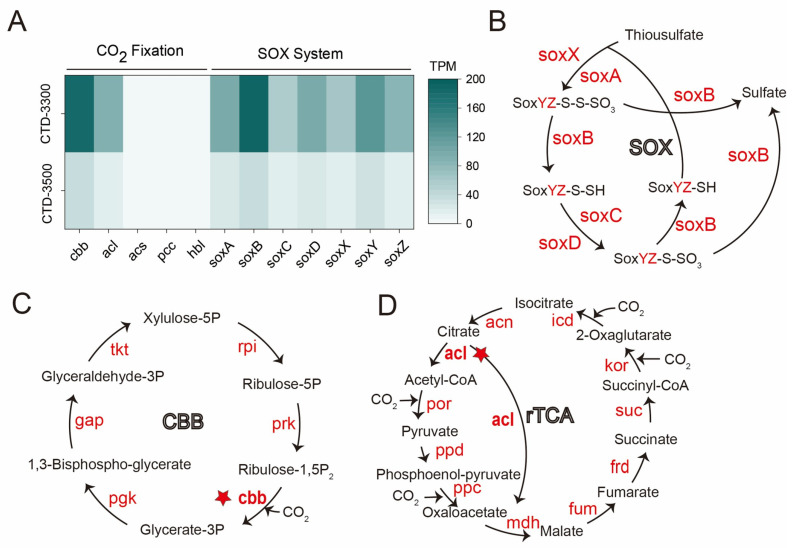
Carbon fixation and thiosulfate oxidation pathways and the abundances of their key enzyme genes in 85° E samples. (**A**) Heat map shows the normalized abundance of key enzyme genes. (**B**) SOX pathway: L-cysteine S-thiosulfotransferase (soxAX), S-sulfosulfanyl-L-cysteine sulfohydrolase (soxB), sulfane dehydrogenase (soxC), S-disulfanyl-L-cysteine oxidoreductase (soxD), and sulfur-oxidizing protein (soxYZ). (**C**) Calvin-Benson-Bassham (CBB) cycle: ribulose-1,5-bisphosphate carboxylase (key gene, cbb), phosphoglycerate kinase (pgk), glyceraldehyde-3-phosphate dehydrogenase (gap), transketolase (tkt), ribose 5-phosphate isomerase (rpi), and phosphoribulokinase (prk). (**D**) Reductive tricarboxylic acid (rTCA) cycle: ATP-citrate lyase (key gene, acl), pyruvate ferredoxin oxidoreductase (por), pyruvate, orthophosphate dikinase (ppd), phosphoenolpyruvate carboxylase (ppc), malate dehydrogenase (mdh), fumarate hydratase (fum), NADH-dependent fumarate reductase (key gene, frd), succinyl-CoA synthetase (suc), 2-oxoglutarate/2-oxoacid ferredoxin oxidoreductase (kor), isocitrate dehydrogenase (idh), and aconitate hydratase (acn). The red star marks key enzyme genes.

Seawater and hydrothermal plume water samples were collected at CTD 01 station using an SBE-32 carousel water sampler. 4 L volumes of water samples were collected at depths of 3000 m, 3200 m, 3250 m, 3300 m, 3350 m, 3400 m, and 3500 m (Table S1). The samples were filtered through 0.22 μm pore size polycarbonate membranes (47 mm diameter, Millipore, Darmstadt, Germany) to collect particulates. The filter membranes and filtrate were stored at −80 °C for subsequent genomic and geochemical analysis, respectively. Fresh volcanic glass fragments and volcaniclastic material were grabbed at TVG 01 station and TVG 06 station (Figure 1B-2,B-4). Surface sediments were collected at GC 01 station, approximately 5 km away from the CTD 01 station (Figure 1A). The sedimentary core was carefully subdivided into thin layers at intervales of 2–3 cm and stored at −80 °C for subsequent genomic and geochemical analysis (Table S1). Dissolved inorganic carbon (DIC) and its δ^13^C_DIC_ isotope in water samples were measured using a Delta V advantage isotope mass spectrometer (Thermo Fisher Scientific, Waltham, MA, USA). Dissolved organic carbon (DOC) was quantified with a TOC-L CPH (SHIMADZU, Kyoto, Japan). Extractable petroleum hydrocarbons (C_10_–C_40_) were measured by gas chromatograph (SHIMADZU, Kyoto, Japan). The total organic carbon (TOC) in sediments was determined using the combustion oxidation nondispersive infrared absorption method.

### 2.3. DNA Extraction, 16S rRNA Gene Sequencing and Analysis

DNA was extracted from particulate samples and sediment core samples using the DNeasy Powersoil Pro kit (QIAGEN, Valencia, CA, USA) following the manufacturer’s instructions. The DNA quality was assessed by NanoDrop2000 (Thermo Fisher Scientific, Waltham, MA, USA), and concentration was measured by Quantus Fluorometer (Promega, Madison, WI, USA). The V4 region of the 16S rRNA gene was amplified with the primer pair 515F (5′-GTGCCAGCMGCCGCGGTAA-3′) and 806R (5′-GGACTACHVGGGTWTCTAA-3′) [34]. PCR reactions were performed in 20 μL reactions, each containing 0.8 μL (5 μM concentration) of forward and reverse primers, 10 μL Pro Taq, and 2 μL of genomic community DNA as a template. Each sample was amplified in triplicate and mixed after PCR amplification to reduce potential amplification bias. Thermal cycling conditions were as follows: initial denaturation at 95 °C for 3 min, followed by 29 cycles of 95 °C for 30 s, 53 °C for 30 s, and 72 °C for 45 s, with final extension at 72 °C for 10 min. The amplified gene libraries were sequenced using the Hiseq PE250 sequencing platform (Illumina, San Diego, CA, USA) to obtain raw sequences. All bioinformatics analyses were performed using QIIME2 [35]. Raw demultiplexed sequences were denoised and dereplicated using DADA2 [36]. The taxonomic assignment of amplicon sequence variants (ASVs) was performed using the SILVA 138 database [37]. Additionally, 14 publicly available 16S rRNA datasets generated from Aurora and Polaris hydrothermal plume samples (Table S1) were downloaded from the European Nucleotide Archive under project number PRJNA82185.

### 2.4. Metagenomic Sequencing and Analysis

Metagenomic sequencing analysis was performed on particulate samples collected from depths of 3300 m and 3500 m at CTD 01 station. The environmental DNA was fragmented to construct a gene library, which was then sequenced using the Illumina Novaseq 6000 platform (Illumina, San Diego, CA, USA). The raw reads were trimmed and quality filtered using fastp software (V. 0.23.0) [38]; clean reads were then assembled using Megahit [39]. The metagenomic assemblies were analyzed with Prodigal v.2.6.3 to predict protein coding sequences [40] and annotated with the Kyoto Encyclopedia of Genes and Genomes (KEGG) database [41] using the Blastp program (with an e-value cut-off of 1 × 10^−5^) [42]. To compare differences in gene abundance between samples, transcripts per million (TPM) was employed as a normalization method to eliminate the effect of gene length and sequencing depth on the results [43].

### 2.5. Metagenomic Binning and Metagenome-Assembled Genome (MAG) Classification

Contigs larger than 1000 bp from the final assemblies were binned into draft MAGs with Metabat 2 [44], and the completeness and contamination of bins were evaluated by CheckM [45]. The high- and medium-quality MAGs (completeness ≥ 50% and contamination ≤ 10%) were subsequently subjected to downstream analysis. A dereplication was conducted using FastANI, based on a 95% average nucleotide identity (ANI) [46], and the percentage of conserved proteins (POCP) analysis was employed to ascertain whether the MAGs belonged to different genera (<50%) [47]. MAGs were classified using the GTDB-T classify workflow [48] and with the NCBI taxonomy browser. Use Prodigal v.2.6.3 to predict protein coding sequences [40], and functional annotations were conducted based on comparisons with KEGG [41], Pfam, and P450 databases [49].

## 3. Results

### 3.1. Physicochemical Characteristics of Water Column and Sediments

The concentration of DIC ranged from 9. 60 to 28.57 mg/L, with δ^13^C_DIC_ varying between −9.04 and −0.73‰. Notably, at depths of 3300 m and 3400 m, the highest and lowest values of δ^13^C_DIC_ were observed, which occurred in parallel with the highest and lowest concentrations of DIC, respectively (Figure 2B). The concentrations of DOC and C_10_–C_40_ ranged from 1.01 to 1.43 mg/L and 0.54 to 0.59 mg/L, with the lowest concentration of them both observed at 3300 m (Figure 2C). The TOC content in the sediment samples ranged from 0.45% to 1.05%, and a downcore decreasing trend was observed (Figure 3E).

### 3.2. Microbial Diversity and Its Correlation with Environmental Factors

The sequences were rarefied to the lowest amplification sequence number (120,713), obtained 844,911 valid reads, which were clustered into 3619 amplicon sequence variants (ASVs). The results showed that the dominant taxa in the 85° E hydrothermal plume influence zone were as follows: Gammaproteobacteria (21.2–86.0%), with the highest relative abundance at 3500 m; Campylobacteria (1.0–51.9%), peaking at 3300 m and the lowest in background seawater (3000 m); and Desulfuromonadia (0.1–45.3%), Alphaproteobacteria (1.4–15.8%), and Nitrososphaeria (1.0–14.4%) (Figure 3A). At the genus taxonomic level, the dominant taxa were as follows: *Sulfurimonas* (1.0–51.8%) and *SUP05_cluster* (0.9–7.6%), both maximized at 3300 m with decreasing abundance toward shallower/deeper depths; *Alcanivorax* (up to 82.5%), which had the highest relative abundance at 3500 m (After normalization by RPKM in metagenomic analysis, the highest abundance reached 82.9%, Figure S1); and *Mycobacterium* (up to 11.5%), *Acinetobacterium* (up to 8.2%), and *Alteromonas* (up to 7.1%) (Figure 3B).

As a comparison, the samples from the Aurora and Polaris hydrothermal plumes were significantly enriched *Sulfurimonas* (0–20.9% and 2.2–67.5%, respectively) and *SUP05_cluster* (5.0–13.6% and 1.7–24.3%, respectively) [24], which were sort of similar to those in the 85° E hydrothermal plume. Whereas, notably, *Alcanivorax* was virtually undetectable in the Aurora nor Polaris plumes, contrasting with the high relative abundance in the 85° E hydrothermal plume (Figure 3C). Furthermore, in the 85° E sediments, *Alcanivorax* exhibited a high relative abundance (up to 6.5%) in the upper sedimentary layers, decreasing with depth (Figure 3D). The abundance of *Alcanivorax* and the content of TOC in sediments have a significant positive correlation (Figure 3E, r = 0.75, *p* < 0.05).

### 3.3. Carbon Fixation and Sulfur Oxidation Potential

The metagenomic sequencing data of the hydrothermal plume samples collected at water depths of 3300 m and 3500 m were analyzed, and complete SOX systems (thiosulfate oxidase systems) genes were identified (Figure 4B). Furthermore, the genes encoding for multiple carbon fixation pathways, including the Calvin–Benson–Bassham (CBB) cycle, Reductive tricarboxylic acid (rTCA) cycle, Wood–Ljungdahl (WL) pathway, 3-hydroxypropionate (3HP) cycle, Hydroxypropionate/Hydroxybutyrate (HP/HB) cycle, and Dicarboxylate/Hydroxybutyrate (DC/HB) cycle (Figure 4C,D and Figure S2). After TPM (transcripts per million) normalization, the gene abundance results showed that the key enzyme gene (cbb) involved in the CBB cycle was significantly more abundant than those of other carbon fixation pathways, closely followed by the rTCA cycle (acl). In addition, the abundance of key enzyme genes associated with the CBB, rTCA, and sulfur oxidation pathways was significantly higher at 3300 m than at 3500 m (Figure 4A).

### 3.4. Organic Carbon Degradation Potential and Alcanivorax-Associated MAGs

**Figure 5:**
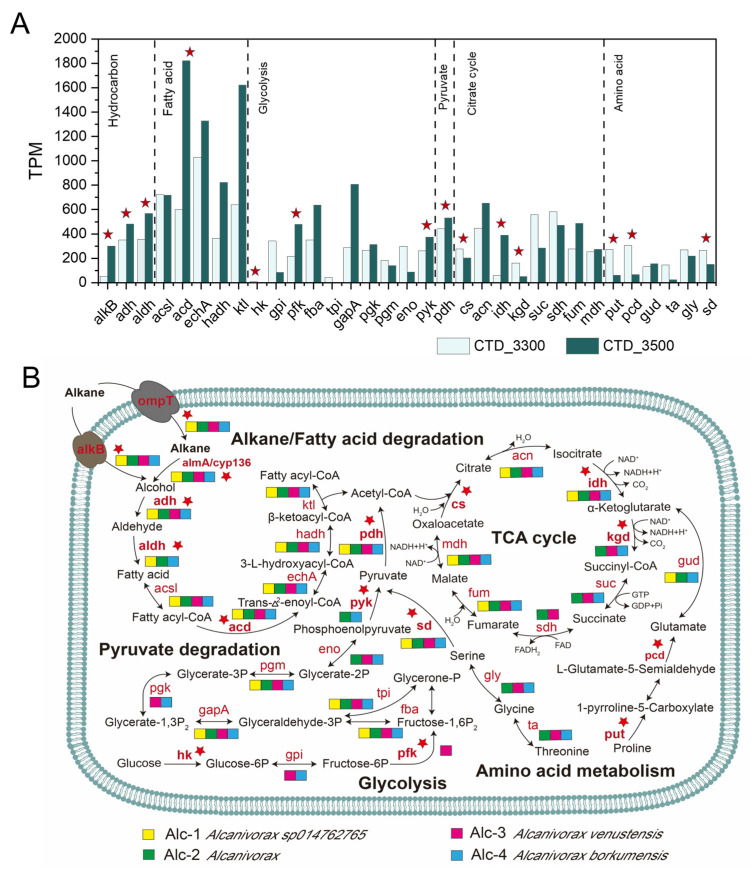
Metabolic potential for organic carbon degradation in 85°E hydrothermal samples. (**A**) Normalized abundance of key enzyme genes for organic carbon degradation metabolism pathways. (**B**) Organic carbon degradation potential in MAGs of *Alcanivorax*; alkane/fatty acid degradation, genes: outer membrane transport protein (key gene, ompT), alkane monooxygenase (key gene, alkB), flavin-binding monooxygenase (almA), cytochrome P450 (key gene, cyp136), alcohol dehydrogenase (adh), aldehyde dehydrogenase (NAD+) (aldh), acyl-CoA synthetase (acsl), acyl-CoA dehydrogenase (keygene, acd), enoyl-CoA hydratase (echA), 3-hydroxyacyl-CoA dehydrogenase (hadh), β-Ketothiolase (ktl); glycolysis, genes: hexokinase (Key gene, hk), glucose-6-phosphate isomerase (gpi), 6-phosphofructokinase (pfk), fructose-bisphosphate aldolase (fba), triosephosphate isomerase (tpi), glyceraldehyde 3-phosphate dehydrogenase (gapA), phosphoglycerate kinase (pgk), phosphoglycerate mutase (pgm), enolase (eno), pyruvate kinase (pyk); pyruvate degradation, genes: pyruvate dehydrogenase (key gene, pdh); TCA cycle, genes: citrate synthase (key gene, cs), aconitate hydratase (acn), isocitrate dehydrogenase (key gene, idh), alpha-ketoglutarate dehydrogenase (key gene, kgd), succinyl-CoA synthetase (suc), succinate dehydrogenase (sdh), fumarate hydratase (fum), malate dehydrogenase (mdh); amino acid metabolism, genes: proline dehydrogenase (put), 1-pyrroline-5-carboxylate dehydrogenase (key gene, pcd), glutamate dehydrogenase (gud), threonine aldolase (ta), glycine hydroxymethyltransferase (gly), and L-serine dehydratase (key gene, sd). Red stars denote key enzyme genes.

The genes encode for multiple organic carbon degradation metabolic pathways, including those for alkane/fatty acid degradation, glycolysis, pyruvate degradation, amino acid degradation, and the tricarboxylic acid cycle (TCA). The results of normalized abundance of key enzyme genes revealed that the degradation potential for alkanes (alkB, adh, and aldh) exhibited higher potential than that for glycolysis (hk, pfk, and pyk), pyruvate metabolism (pdh), and amino acid degradation (put, pcd, and sd). Except for amino acid degradation, the abundance of other organic carbon degradation-related enzyme genes at 3300 m was significantly lower compared to 3500 m (Figure 5A). We assembled the dominant *Alcanivorax* genome and analyzed its potential for degrading organic carbon.

We assembled the dominant *Alcanivorax* genome and analyzed its organic carbon degradation potential. A total of four *Alcanivorax*-related MAGs were recovered (Alc-1 to Alc-4), with a completeness of ≥50% and a contamination of <5% (Table S2). Alc-1, Alc-3, and Alc-4 were identified as *Alcanivorax* sp014762765, *Alcanivorax* venustensis, and *Alcanivorax* borkumensis, respectively. Alc-2 was identified at the genus *Alcanivorax*. Pairwise PCOP analysis between each of the *Alcanivorax*-related MAGs (Alc-1 to Alc-4) and the cultivated species *Alcanivorax* borkumensis, revealed protein sequence similarities exceeding 50% in all cases. This indicated that they belong to the same genus (Figure S3). All MAGs contained genes encoding alkane transport (ompT) and alkane monooxygenase (alkB), alcohol dehydrogenase (adh), and cytochrome P450 (cyp136), which are involved in the degradation of short-to-medium chain alkanes (C_5_–C_17_). Putative flavin-dependent monooxygenases responsible for the degradation of long alkanes (almA) (C_32+_) were encoded in all MAGs. For fatty acid metabolism, fatty acids are broken down via β-oxidation to acetyl CoA, which then enters the tricarboxylic acid (TCA) cycle. The initial step in the TCA cycle is the condensation of acetyl CoA with oxaloacetate, catalyzed by citrate synthase (cs), to form citrate. In this cycle, each molecule of acetyl CoA undergoes four dehydrogenation reactions, producing three molecules of NADH+H+ and one molecule of FADH_2_, accompanied by two decarboxylation reactions releasing CO_2_. However, the gene encoding citrate synthase, which is one of the key enzymes in the TCA cycle, was not detected in any of the MAGs studied. All MAGs encode partial genes for glycolysis and amino acid metabolism but lack the first enzyme-coding gene of glycolysis (hexokinase, hk) (Figure 5B).

## 4. Discussion

### 4.1. Chemosynthetic Taxa and Energy Metabolism Potential

*Sulfurimonas* and *SUP05_cluster* are typically the dominant taxa found in basalt-hosted hydrothermal plumes, fixing carbon via autotrophic sulfur oxidation, and serving as the cornerstone of the hydrothermal ecosystem [24,50,51]. In the 85° E hydrothermal plume, their relative abundance covaried with turbidity and peaked at 3300 m, coinciding maxima in DIC and δ^13^C_DIC_. Previous studies have shown that the δ^13^C_DIC_ values decrease with water depth due to heterotrophic remineralization of sinking particulate organic matter [52,53,54]. It was thus inferred that the elevated δ^13^C_DIC_ at 3300 m indicates hydrothermal input of ^13^C-enriched DIC. Hydrothermal inputs generated a multi-source carbon mixing signature, influencing the overall concentration of DIC and its isotopic composition.

Concurrently, the input of reduced compound such as sulfides from hydrothermal fluids stimulated proliferation of autotrophic groups such as *Sulfurimonas* and *SUP05_cluster*. Campylobacteria (e.g., *Sulfurimonas*) are typically found in low-oxygen and high-sulfide environments, Gammaproteobacteria (e.g., *SUP05_cluster*) are typically found in high-oxygen and low-sulfide environments [55,56]. The relative abundance of *Sulfurimonas* was lower than that of the *SUP05_cluster* at the depth of 3000 m (near background seawater) in the 85° E hydrothermal plume. At other depths, the relative abundance of *Sulfurimonas* was higher than that of the *SUP05_cluster*. It implied that the oxygen content in the water closer to the background seawater was higher than in the hydrothermal plume layer, while the sulfide content was lower. Therefore, the relative abundance of *SUP05_cluster* was higher than *Sulfurimonas* in the background seawater.

Metagenomic analysis indicated that microbial carbon fixation in the 85° E hydrothermal plume may mainly be via the CBB cycle, with the rTCA cycle serving as a secondary pathway, consistent with findings in global deep-sea hydrothermal vents reported by others [57,58]. Chemolithoautotrophic microbes using the CBB cycle for carbon fixation are mainly classified into Alphaproteobacteria, Gammaproteobacteria, Betaproteobacteria, Firmicutes, and Chloroflexi [59]. In the 85° E hydrothermal plume, the *SUP05_cluster* may have used the CBB cycle for carbon fixation. The metabolic potential for carbon fixation and sulfur oxidation at 3300 m was significantly higher than at 3500 m, indicating intense chemosynthetic activity within the center layer of nonbuoyant hydrothermal plume. The chemosynthetic carbon fixation process exhibited high consistency with global basalt-hosted hydrothermal systems [26]. This finding provides data support for global hydrothermal system models, further enriching our understanding of the operating mechanisms of hydrothermal ecosystems.

### 4.2. Explosive Volcanism as a Potential Driver of Heterotrophic Bacteria Bloom Represented by Alcanivorax

*Alcanivorax*, known for its ability to degrade hydrocarbons, occupied a very high abundance in oil-contaminated marine ecosystems across the globe earth [60,61,62]. However, the abundance of *Alcanivorax* at the water depth of 3500 m in the 85° E hydrothermal plume reached 82.9% (Figure 3B), which is even higher than that documented in oil spills. The significant positive correlation between the relative abundance of *Alcanivorax* and the content of TOC in surface sediments, indicates that the distribution and abundance of *Alcanivorax* are closely related to the organic carbon (Figure 3E). It is known that young crust and sediments on the mid-ocean ridges lead no potential oil or gas reservoirs, especially in the axial valley [63,64,65]. Thus, it can be suggested that the hydrocarbons feeding high abundance *Alcanivorax* should come from other than oil-contaminated environments.

As the ultra-slow spreading ridge, the Gakkel Ridge has ample time to accumulate terrestrial and glacially transported sediments, resulting in the formation of a relatively thick sedimentary layer rich in organic matter [66,67,68]. Our cruise confirmed that the TOC content of surface sediment can reach up to 1.05% (JASMInE, 2021). However, *Alcanivorax* was barely detectable in hydrothermal plumes of the Aurora or Polaris field located in the western and central end of the Gakkel Ridge, respectively, where the volcanically-hosted hydrothermal systems or seabed sedimentary conditions show almost no difference compared to 85° E [11,24,68,69]. This implies that the excess supply of hydrocarbons for the remarkably abundant *Alcanivorax* in the 85° E hydrothermal plume could be provided by the seabed organic-rich sediment, but not through the process of submarine hydrothermal circulation.

Through the near-bottom optical survey, we found fresh volcaniclastic deposits (>10 cm thick) on the flank of Oden volcano (2–3 km south-east of our sampling site), indicating that Oden volcano may have experienced a relatively intense explosive eruption shortly before the cruise survey. This contrasts with the effusive-dominated volcanism at the Aurora hydrothermal field [10,12]. Unlike the effusive volcanic activity, explosive volcanic eruptions often involve a rapid release of energy, causing severe disturbance to the local oceanic crust, overlying sediments, and water bodies [70].

The high abundance of *Alcanivorax* has been reported in hydrothermal plumes over the Tonga Arc, where active volcanism occurs. It was hypothesized that volcanic-induced hydrocarbon leakage fuels these *Alcanivorax* populations [71]. Additionally, high abundance of *Alcanivorax* was also found in the surface sediments approximately 5 km north-east of our sampling site. Therefore, it is reasonable to speculate that the explosive volcanic eruption triggered the release of buried organic carbon into the overlying water column and entrained by the hydrothermal plume. This process most likely caused the surge in *Alcanivorax* in the 85° E hydrothermal plume.

### 4.3. Effects on Carbon Cycling in the Deep Ocean

**Figure 6:**
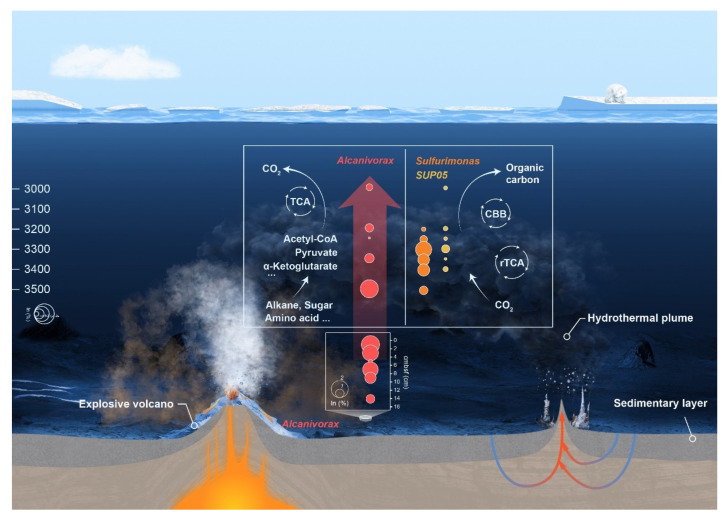
Dominant microbial genera and their carbon conversion potential in 85° E hydrothermal plume of the explosive volcanic zone at Gakkel Ridge.

In the Gakkel Ridge 85° E ecosystem, chemoautotrophic populations facilitate the conversion of inorganic carbon released from volcanic and hydrothermal activities into organic carbon through metabolic pathways, including the CBB cycle and the rTCA cycle. At a depth of 3500 m (near the seafloor), there was a high abundance of genes related to the degradation of organic carbon, including hydrocarbons, sugars, and amino acids. Genes related to hydrocarbon degradation were more abundant than that for others. The variation in dissolved C_10_–C_40_ compounds and DOC content with depth showed a consistent pattern, suggesting that DOC may consist mainly of petroleum hydrocarbons. We propose that explosive volcanic eruption, as a violent geological phenomenon, has the capacity to disrupt the stability of sedimentary layers, leading to the release of previously sequestered hydrocarbons into the adjacent seawater (Figure 6). This process perturbs the balance between burial and release of deep-sea carbon along mid-ocean ridges. The high selective pressure exerted by hydrocarbons from sedimentary layers leads to rapid shifts in microbial populations in the seawater, thereby reducing the diversity of the microbial community [72]. At this stage, the metabolic processes of microorganisms such as *Alcanivorax* become particularly important, as they can accelerate the mineralization of these organic carbon compounds. The four MAGs of *Alcanivorax* from 85° E hydrothermal plume not only contain encode alkB and cytochrome P450 genes for the degradation of short- and medium-chain hydrocarbons but also encode the almA gene capable of degrading long-chain hydrocarbons. AlkB is a transmembrane metal enzyme, in the initial stage of microbially mediated alkane degradation, facilitates the conversion of water-insoluble straight-chain alkanes into more biodegradable alcohols [73]. AlmA is capable of oxidizing alkanes at the terminal end and initiating the degradation of long-chain alkanes through subterminal oxidation [74]. Subterminal oxidation is a more effective method of attacking long-chain hydrocarbon molecules, initiating and accelerating the complete degradation process. All MAGs of *Alcanivorax* in our research have the potential to complete the final step of hydrocarbon degradation, which involves converting organic carbon into CO_2_ through the TCA cycle (Figure 6). Lin et al. [75] also demonstrated that *Alcanivorax* can mineralize medium-chain alkanes to CO_2_.

The explosive volcanism at the 85° E segment was a regional, transient geological event that caused the release of organic matter from sediments and reshaped the structure of microbial communities. However, given the vast length of global mid-ocean ridges (—65,000 km) and the numerous volcanic activities that remain undiscovered [76,77], the explosive volcanic effects represent a universal and non-negligible biogeochemical processes in the entire ocean and throughout Earth’s history. The focus of carbon cycling research has long been on Earth’s surface systems, whereas the lithosphere is the largest carbon reservoir [78,79]. Up to now, there is still a lack of in-depth understanding of the mechanisms of the deep carbon cycling, especially on the patterns of carbon exchange between the oceanic crust lithosphere, hydrosphere and biosphere [80,81].

This study highlights an often-overlooked phenomenon: intense geological activity may trigger the release of carbon from deep-sea sedimentary layers serving as vital carbon sinks. The released carbon re-enters the fast carbon cycle, with microbes playing a significant role in the process. This insight enhances our comprehensive understanding of the operating mechanisms of the deep ocean carbon cycling, which is influenced by the interplay of geological events, hydrothermal systems, and microbiomes.

## 5. Conclusions

The explosive volcanism and hydrothermal activity along the eastern segment of the Gakkel Ridge provided a harsh and dynamic habitat for life. We found that the main chemosynthetic groups and carbon fixation pathways in 85° E are consistent with those of global basaltic hydrothermal systems. It is noteworthy that *Alcanivorax* was significantly enriched in both the hydrothermal plume near the seafloor and the sediments, potentially degrading hydrocarbons and releasing CO_2_. We speculate that the proliferation of heterotrophic bacteria is associated with the release of sedimentary organic matter into the overlying water column as a result of recent explosive volcanic activity, based on the evidence of volcanic eruptions observed on the seafloor. These findings support the hypothesis that local geological events shape microbial communities. Our study revealed the microbial diversity and metabolic mechanisms of this unique environment, providing new insights into the interactions between geological activities and microbial communities, and highlighted that heterotrophic microbial communities play a significant role in accelerating the slow carbon cycle within geologically active areas. Future research could consider monitoring of geological events and conducting periodic microbial sampling to further investigate the long-term interactions between geological activities and the carbon cycle.

## Data Availability

All the raw 16S rRNA and metagenomic data generated in this study are publicly available in the NCBI BioProject with the accession PRJNA1279799.

## Supplementary Materials

The following supporting information can be downloaded at: https://www.mdpi.com/article/10.3390/biology14081036/s1, Figure S1: Relative abundance of *Alcanivorax* after normalization by RPKM (Reads Per Kilobase Million) based on metagenomic results; Figure S2: Carbon fixation pathways in 85° E samples. (A) WL cycle; genes: acetyl-CoA synthase (key gene, acs), anaerobic carbon-monoxide dehydrogenase catalytic (coo), formate dehydrogenase (fdh), formate-tetrahydrofolate (fhs), methylenetetrahydrofolate dehydrogenase (fol), methylenetetrahydrofolate reductase (met). (B) 3HP cycle; genes: propionyl-CoA carboxylase (key gene, pcc), methylmalonyl-CoA mutase (mut), malyl-CoA (mcl), 3-methylfumaryl-CoA hydratase (meh). (C) HP/HB and DC/HP cycle; genes: 4-hydroxybutyrate-CoA ligase (key enzyme, hbl), acetyl-CoA C-acetyltransferase (key gene, acat), methylmalonyl-CoA/ethylmalonyl-CoA epimerase (mcee), methylmalonyl-CoA mutase (mut), pyruvate ferredoxin oxidoreductase (por), pyruvate, orthophosphate dikinase (ppd), phosphoenolpyruvate carboxylase (ppc), phosphoenolpyruvate carboxylase (ppc), malate dehydrogenase (mdh), fumarate hydratase (fum); Figure S3: The PCOP analysis of MAGs of *Alcanivorax* in this study; Table S1: Samples analyzed in this study; Table S2: The statistics to the MAGs of *Alcanivorax* retrieved in this study.
