# Supplementary material for: High-Abundance Heterotrophic Bacteria Inhabit the 85° E Hydrothermal Plume of the Explosive Volcanic Zone at Gakkel Ridge, Arctic Ocean
Source: Biology (Basel). 2025 Aug 12;14(8):1036. doi: 10.3390/biology14081036 (PMC12383903; doi:10.3390/biology14081036)
Supplement: Supplementary file 1 [file biology-14-01036-s001.zip › biology-3782903-supplementary.pdf]

Supplementary material

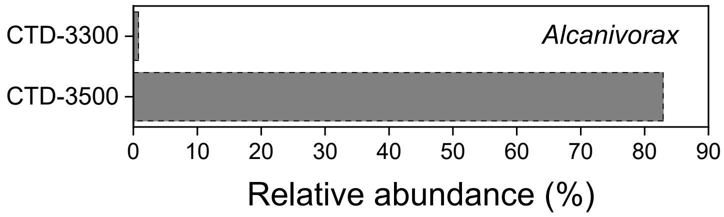

Figure S1 Relative abundance of *Alcanivorax* after normalization by RPKM (Reads Per Kilobase Million) based on metagenomic results.

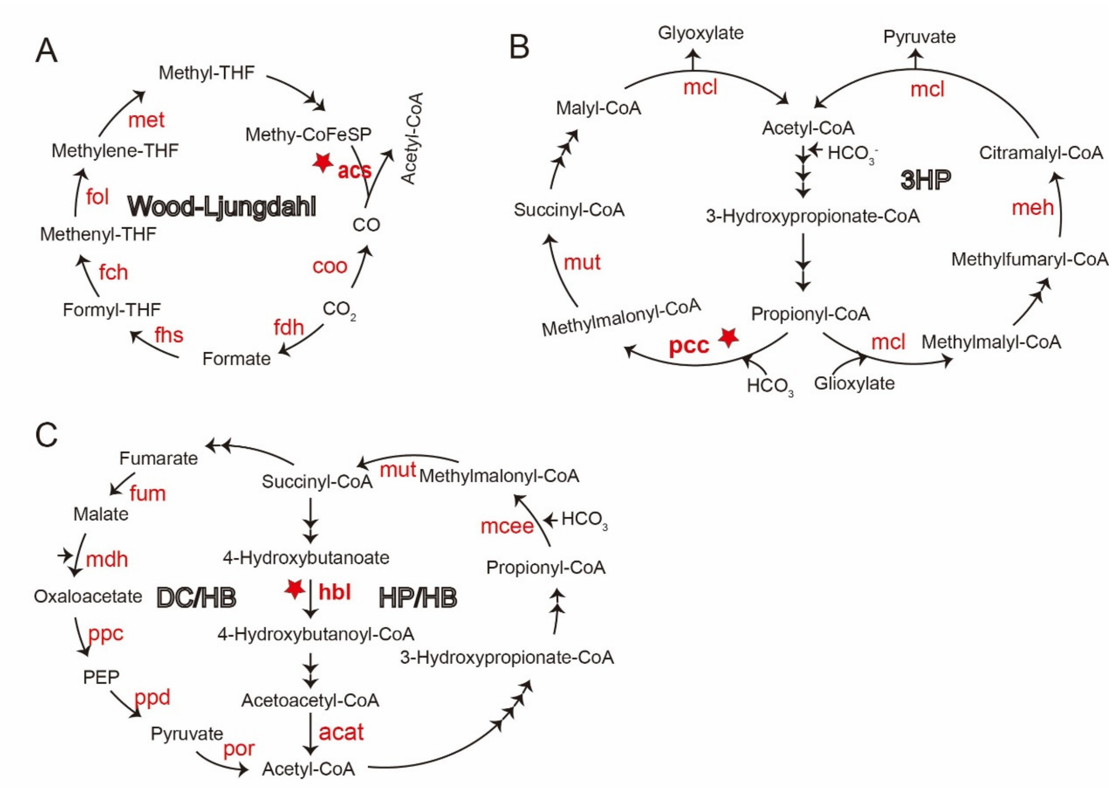

Figure S2 Carbon fixation pathways in 85°E samples. (A) WL cycle, genes: acetyl-CoA synthase (key gene, acs), anaerobic carbon-monoxide dehydrogenase catalytic (coo), formate dehydrogenase (fdh), formate-tetrahydrofolate (fhs), methylenetetrahydrofolate dehydrogenase (fol), methylenetetrahydrofolate reductase (met). (B) 3HP cycle; genes: propionyl-CoA carboxylase (key gene, pcc), methylmalonyl-CoA mutase (mut), malyl-CoA (mcl), 3-methylfumaryl-CoA hydratase (meh). (C) HP/HB and DC/HP cycle; genes: 4-hydroxybutyrate-CoA ligase (key enzyme, hbl), acetyl-CoA C-acetyltransferase (key gene, acat), methylmalonyl-CoA/ethylmalonyl-CoA epimerase (mcee), methylmalonyl-CoA mutase (mut), pyruvate ferredoxin oxidoreductase (por), pyruvate, orthophosphate dikinase (ppd), phosphoenolpyruvate carboxylase (ppc), phosphoenolpyruvate carboxylase (ppc), malate dehydrogenase (mdh), fumarate hydratase (fum).

	Alc-1	Alc-2	Alc-3	Alc-4	*A. borkumensis*
Alc-1	NA				
Alc-2	79.84	NA			
Alc-3	57.37	62.00	NA		
Alc-4	64.37	70.75	62.11	NA	
A. borkumensis	70.01	77.96	67.69	86.26	NA

Figure S3 The PCOP analysis of MAGs of *Alcanivorax* in this study.

Table S1 Samples analyzed in this study.

Area	Sample	Station	Sampling	Latitude	Longitude	Depth(m)	Sample
Gakkel 85°E	CTD01-3000	CTD01	07.08.2021	85.63	85.14	3000	Above
	CTD01-3200	CTD01	07.08.2021	85.63	85.14	3200	Above
	CTD01-3250	CTD01	07.08.2021	85.63	85.14	3250	Above
	CTD01-3300	CTD01	07.08.2021	85.63	85.14	3300	Plume
	CTD01-3350	CTD01	07.08.2021	85.63	85.14	3350	Plume
	CTD01-3400	CTD01	07.08.2021	85.63	85.14	3400	Plume
	CTD01-3500	CTD01	07.08.2021	85.63	85.14	3500	Plume
	GC01-1	GC01	24.08.2021	85.66	85.72	3973	Sediment
	GC01-3	GC01	24.08.2021	85.66	85.72	3973	Sediment
	GC01-5	GC01	24.08.2021	85.66	85.72	3973	Sediment
	GC01-7	GC01	24.08.2021	85.66	85.72	3973	Sediment
	GC01-9	GC01	24.08.2021	85.66	85.72	3973	Sediment
	GC01-12	GC01	24.08.2021	85.66	85.72	3973	Sediment
	GC01-14	GC01	24.08.2021	85.66	85.72	3973	Sediment
	GC01-16	GC01	24.08.2021	85.66	85.72	3973	Sediment
	GC01-18	GC01	24.08.2021	85.66	85.72	3973	Sediment
	GC01-20	GC01	24.08.2021	85.66	85.72	3973	Sediment
	GC01-22	GC01	24.08.2021	85.66	85.72	3973	Sediment
	GC01-24	GC01	24.08.2021	85.66	85.72	3973	Sediment
Gakkel Polaris	Po-2336	PS101-	28.09.2016	86.96	55.93	2336	Above
	Po-2492	PS101-	05.10.2016	86.97	55.95	2492	Above
	Po-2640	PS101-	03.10.2016	86.96	55.68	2640	Plume
	Po-2716	PS101-	05.10.2016	86.96	55.83	2716	Plume
	Po-2846	PS101-	28.09.2016	86.96	55.96	2846	Plume
	Po-3006	PS101-	28.09.2016	86.95	55.91	3006	Below
	Po-3107	PS101-	23.09.2016	86.95	55.58	3107	Below
Gakkel Aurora	Au-2000	PS86-57	22.07.2014	82.90	-6.23	2000	Above
	Au-2500	PS86-66	25.07.2014	82.90	-6.23	2500	Above
	Au-2900	PS86-69	26.07.2014	82.90	-6.27	2900	Above
	Au-3290	PS86-23	13.07.2014	82.90	-6.25	3290	Plume
	Au-3400	PS86-55	22.07.2014	82.90	-6.25	3400	Plume
	Au-3500	PS86-57	22.07.2014	82.90	-6.23	3500	Below
	Au-3575	PS86-66	25.07.2014	82.90	-6.23	3575	Plume

Table S2 The statistics to the MAGs of *Alcanivorax* retrieved in this study.

MAG	Taxonomy	Comp. (%)	Cont. (%)	Genome size（bp）	GC (%)	contigs	N50 (bp)
Alc-1	*Alcanivorax sp014762765*	50	0	2535948	58.66	375	7905
Alc-2	*g_Alcanivorax*	83.9	3.52	3106441	59.14	584	6824
Alc-3	*Alcanivorax venustensis*	70.85	3.45	2070836	66.75	389	6882
Alc-4	*Alcanivorax borkumensis*	54.31	1.72	2506178	54.98	409	7617
